# Evidence for a bi-partition of the Younger Dryas Stadial in East Asia associated with inversed climate characteristics compared to Europe

**DOI:** 10.1038/srep44983

**Published:** 2017-03-31

**Authors:** Gordon Schlolaut, Achim Brauer, Takeshi Nakagawa, Henry F. Lamb, Jonathan J. Tyler, Richard A. Staff, Michael H. Marshall, Christopher Bronk Ramsey, Charlotte L. Bryant, Pavel E. Tarasov

**Affiliations:** 1 Centre for Ocean Drilling Science (ODS), Japan Agency for Marine-Earth Science and Technology (JAMSTEC), 3173-25 Showa-machi, Kanazawa-ku, Yokohama, 236-0001 Japan; 2 Section 5.2: Climate Dynamics and Landscape Evolution, GFZ German Research Centre for Geosciences, Telegrafenberg, 14473 Potsdam, Germany; 3 Research Centre for Palaeoclimatology, Ritsumeikan University, 1-1-1 Noji-Higashi, Kusatsu, Shiga 525-8577, Japan; 4 Department of Geography, University of Newcaslte upon Tyne, Newcastle upon Tyne, NE1 7RU, UK; 5 Department of Geography and Earth Sciences, Aberystwyth University, Aberystwyth SY23 3DB, UK; 6 Department of Earth Sciences, University of Adelaide, South Australia 5005, Australia; 7 Research Laboratory for Archaeology and the History of Art (RLAHA), University of Oxford, Oxford, OX1 3QY, UK; 8 NERC Radiocarbon Facility (East Kilbride), Scottish Enterprise Technology Park, Rankine Avenue, East Kilbride, Scotland, G75 OQF, UK; 9 Institute of Geological Sciences, Palaeontology Section, Free University Berlin, Malteserstr, 74-100, Haus D, 12249 Berlin, Germany

## Abstract

The Younger Dryas Stadial (YDS) was an episode of northern hemispheric cooling which occurred within the Last Glacial Interglacial Transition (LGIT). A major driver for the YDS climate was a weakening of the Atlantic Meridional Overturning Circulation (AMOC). It has been inferred that the AMOC began to strengthen mid-YDS, producing a bipartite structure of the YDS in records from continental Europe. These records imply that the polar front and westerlies shifted northward, producing a warmer second phase of the YDS in Europe. Here we present multi-proxy data from the sediments of Lake Suigetsu (Japan), as evidence that a related bi-partition of the YDS also occurred in East Asia. Besides showing for the first time that the bi-partition was not limited to the North Atlantic/European region, the data also imply a climatic dipole between Europe and East Asia since the cold-warm characteristics are reversed at Lake Suigetsu. We suggest that changes in eastward moisture transport from the North Atlantic are the primary mechanism by which the teleconnection can be explained.

The Younger Dryas Stadial (YDS) occurred between ≈12.8 and ≈11.6 ka BP and while the exact mechanisms behind the YDS remain enigmatic^1^,^2^,^3^, it is known that a weakening of the Atlantic Meridional Overturning Circulation (AMOC) occurred during this interval^4^, which was a strong driver for the YDS climate. Recent studies show that the AMOC regained some of its strength mid-YDS^5^,^6^,^7^, resulting in a northward shift of the oceanic polar front starting at about 12.3 ka BP^7^. Associated with this shift was an increase in the variability of sea ice cover, due to a negative feedback system between AMOC changes and sea ice extent^5^ resulting in unstable climatic conditions. This climatic change produced a bi-partition of the YDS in European records^5^,^8^,^9^,^10^. For instance, at Lake Kråkenes (Norway), a sudden climatic shift occurred at about 12.15 ka BP, indicating a northward shift of the atmospheric polar front and the westerlies as well as unstable climatic conditions^5^. Furthermore, through linking Lake Kråkenes with Meerfelder Maar (MFM) (Germany), using the Vedde ash as an isochron between the two archives, it was shown that the bi-partition of the YDS occurred earlier in more southerly regions; i.e. that the climatic change was time transgressive from south to north, reflecting the northward shift of the atmospheric polar front^9^. At MFM the bi-partition is recorded at 12.24 ka BP as a sudden increase in spring snowmelt from one year to the next^9^. The climatic change associated with the bi-partition also affected the dust flux in the Greenland ice cores^9^,^11^, with higher values during the first phase of the YDS and lower values during the second, though the mechanism behind this change is uncertain^9^. The transition is not as distinct as in the other records due to a generally high variability, but occurred around 12.1 ka BP.

To investigate whether these North Atlantic changes also affected Asia, a climatically sensitive archive with a robust age model is required. Lake Suigetsu (Japan, 35°35′N, 135°53′E, 0 m above sea level, Fig. 1) represents such an archive in East Asia. It has previously been shown to record changes in the East Asian Monsoon (EAM) system including both the East Asian Winter Monsoon (EAWM) and the East Asian Summer Monsoon (EASM)^12^. Furthermore, it has also been extensively dated^13^ and its chronology is a fundamental part of the IntCal13 radiocarbon calibration dataset^14^. The age model is based on a dual-method varve count^15^,^16^, further constrained by speleothem derived U-Th ages that were modelled onto the varve chronology using the Δ^14^C signals of the respective archives as a linkage^13^. The resulting chronology is denoted ‘SG06_2012_ ka BP’ with the year zero corresponding to A.D. 1950. The SG06_2012_ chronology enables an independent and precise comparison with the Greenland ice cores and with the MFM since the chronologies of these archives are also based on annual laminae counting. Issues with ^14^C dating, such as plateaus in the calibration curve, are thus avoided. For the study presented here, we used the continuous ‘SG06’ composite sediment core^17^. The position of the YDS in the SG06 sediment core was previously determined using pollen, microfacies and diatom data^18^. Combining the position of the YDS boundaries, originally published on a floating varve chronology, with the SG06_2012_ chronology, the YDS began at Lake Suigetsu at 12,780 ± 31 SG06_2012_ yr BP and terminated at 11,593 ± 38 SG06_2012_ yr BP. These ages mean that the YDS was synchronous within dating uncertainties between Suigetsu and the North Atlantic region.

For the study presented here, we conducted a high resolution, multi-proxy analysis of the YDS section of the Lake Suigetsu sediments. Microfacies analysis was applied^16^,^19^ and μXRF measurements were made with a 60 μm resolution^15^. Both methods provided data at sub-annual resolution. Continuous pollen analysis was carried out at 1 cm resolution (corresponding to about 12 years on average). The results of the pollen analysis were then translated into estimates of seasonal and annual temperature and precipitation using the modern analogue technique^20^. δ^15^N measurements on bulk sediment were made at low resolution (≈15 cm for the whole 73 m SG06 profile)^21^.

## Results and Discussion

**Figure 2:**
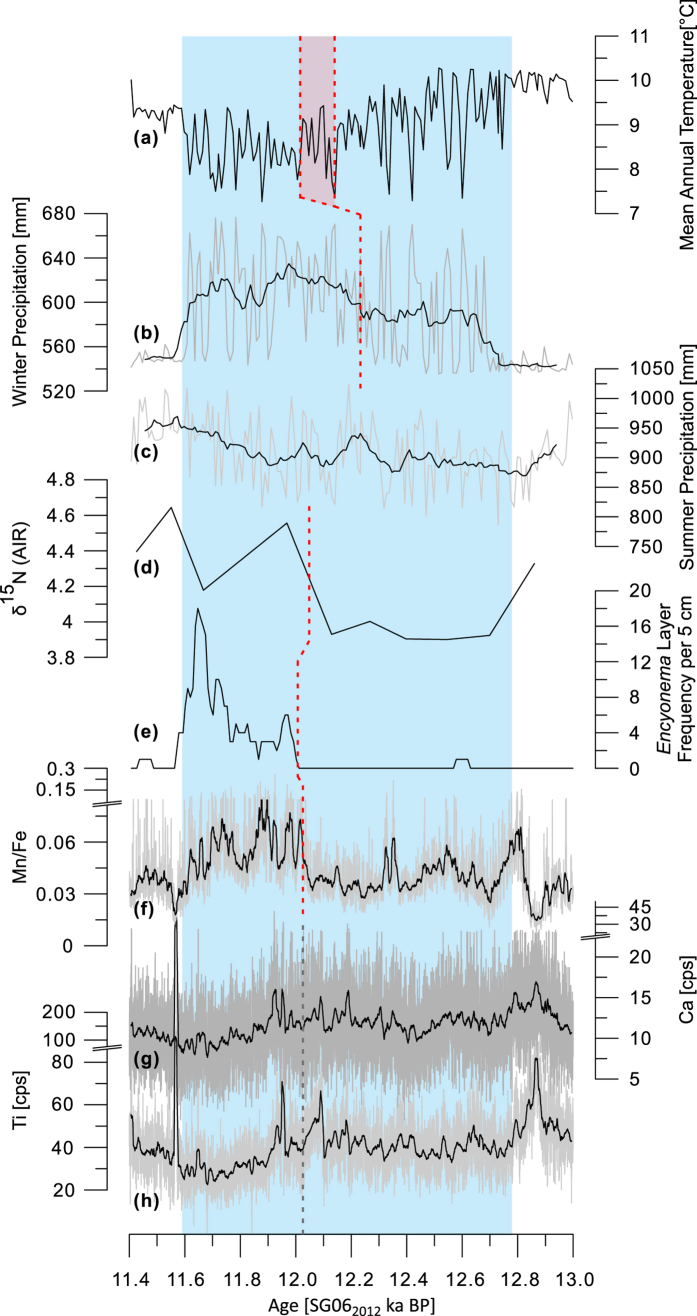
Summary of results from the multi-proxy analysis of the YDS interval of core SG06. In (**b,c**), and (**f**) to (**h**) grey curves are original data; black lines are the moving average ((**b,c**) 11 pt, (**f**) to (**h**) 201 pt); red dashed line marks the point of bi-partition in the respective proxies (see supplement for further details); in (**g**) and (**i**) the trend-signal is overprinted by event layers around the bi-partition, therefore, the point of change is uncertain and a grey dashed line (extrapolated from the Mn/Fe ratio) is used; note that the μXRF curve of K is not shown since it is strongly correlated with Ti (R = 0.74; Fig. S3); light blue background marks the YDS.

All of the employed techniques (microfacies, μXRF, pollen, and bulk isotope analysis) reveal a two-phase character of the YDS at Lake Suigetsu (Fig. 2). The age of the change in the individual proxies varies within the interval 12.22 to 12.01 ka SG06_2012_ BP (Table 1), indicating that the transition between the two YDS phases was coincident with, but not as sudden as at MFM or Lake Kråkenes; it occurred over a longer time frame in which the proxies reacted differentially to the climatic change.

The pollen-based temperature reconstruction shows declining temperatures with a high variability in the first part of the YDS and stable and cool conditions during the second part (Fig. 2a), with no distinguishable difference in character between seasonal and annual reconstructions (Supplementary Fig. S2). It has been shown (for orbital time scales) that temperature changes in Japan are strongly tied to glacial forcing^22^. Therefore the data suggest a southward shift of climate zones caused by an augmentation of the polar cell in the first part of the YDS and a steady state in the second part. The high variability during the first phase of the YDS indicates that the transport of cold polar air was frequently enhanced. In the present-day climate the development of the Okhotsk High can enhance the transport of polar air to northeastern Japan (known as Yamase winds)^23^,^24^. We suggest that a similar, but geographically modified, mechanism was at work during the first phase of the YDS.

The observed cooling also led to a strengthening of the EAWM. The pollen-based precipitation reconstruction shows that winter (October to March) precipitation was enhanced during the YDS, with a further increase occurring in the second phase of the YDS (Fig. 2b), which also resulted in a clear increase in the mean annual precipitation (Fig. S2). These changes can be directly translated into a strengthening of the EAWM^12^ and thus to a cooling of the Siberian/Mongolian air mass. Pollen and μXRF element curves of Ti, K and Ca show that the EASM reacted inversely to the forcing. The pollen-based reconstruction of summer precipitation shows reduced values throughout the YDS (Fig. 2c), indicating a weakening of the summer (April to September) monsoon rainy season. Although the reconstructed summer precipitation does not show the bi-partition of the YDS, a decline in the μXRF element curves of Ti, K and Ca occurs in the second part of the YDS (Fig. 2g,h; Fig. S3). The decline is moderate but statistically significant (Welsh-test yields: p-values < 2.2e-16 for all three elements; data affected by two detrital event layers occurring between 12.10 and 11.93 ka BP were excluded, populations are thus from 12.78–12.10 ka BP and 11.93–11.59 ka BP). The lower values suggest a further weakening of the summer monsoon rainy season in the second phase of the YDS. Since Ti, K and Ca are proxies for detrital inwash into the lake^19^; their decline is indicative of a decrease in catchment erosion and thus a reduction in precipitation (see supplement for further details). Precipitation at Lake Suigetsu is primarily due to the summer monsoon rainy season and to typhoons (Fig. S4). Since the reconstruction of the flood history at Lake Suigetsu shows that the typhoon frequency was reduced throughout the YDS^19^, the moderate decrease in Ti, K and Ca is likely to be due to a weakening of the EASM rainy season in the second part of the YDS. The pollen-based precipitation reconstruction may not be sensitive enough to record this moderate change, especially since the precipitation reconstruction has a higher degree of uncertainty than the temperature reconstruction^20^.

Microfacies and stable isotope data suggest greater turbulence in the lake caused by increased wind stress during the second phase of the YDS. Figure 2d,e show that layers of *Encyonema* diatoms occur in the second part of the YDS and that δ^15^N values are higher. Both proxies have previously been related to an increase in turbulence^17^,^21^ and no indications for diatom dissolution were found during the sediment analysis and therefore the data are not biased in this regard. Hence, the two proxies indicate an increase in wind stress. Greater wind stress is also inferred from the increased Mn/Fe ratio in the second part of the YDS, accompanied by enhanced siderite content (Fig. 2f; Fig. S6). In the case of Lake Suigetsu these changes indicate that the influx of Mn into the lake was increased (see supplement). Wave erosion of the shore as well as geochemical focusing of Mn in deeper parts of the lake due to resuspension of Mn-oxyhydroxides from shallower, oxic parts of the lake are likely explanations, considering that inwash into the lake was reduced as indicated by the μXRF Ti, K and Ca data. We exclude a lake level lowering, which could produce a similar proxy response by increasing turbulence in previously deeper parts of the lake, because the increase in mean annual precipitation (Fig. S2) and the parallel decrease in temperature, and thus evaporation, make a lake level lowering unlikely. The greater wind stress was probably due to the strengthened EAWM and to migratory cyclones and anticyclones developing in spring, since these produce the highest mean monthly wind speeds in the present-day climate^23^,^25^, and because typhoon frequency was reduced^19^. Both mechanisms, EAWM and migratory (anti) cyclones, are strongly tied to the temperature and pressure contrasts between the Siberian/Mongolian and the Pacific air masses and thus support the inference that the bi-partition of the YDS at Lake Suigetsu was brought about by a colder Siberian/Mongolian air mass in the second part of the YDS. However, whether the abrupt change in the Mn/Fe ratio and the *Encyonema* layer frequency is due to a sudden onset of stronger winds or the result of a threshold response to a gradual increase in wind strength over the transition interval remains uncertain.

**Figure 3:**
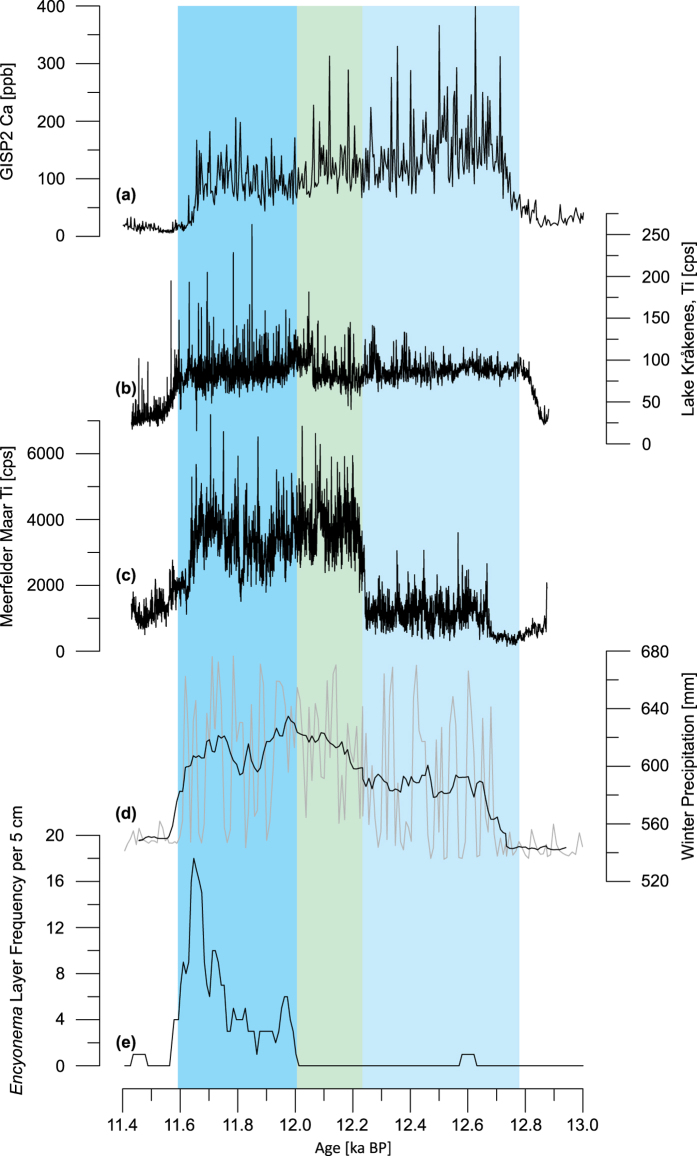
Comparison of (**a**) Greenland GISP2 dust record^11^ (represented by Ca), (**b**) Ti record from Lake Kråkenes^5^ and (**c**) Ti record from MFM^9^ with (**d,e**) results from the study presented here; each plotted on their respective chronologies except (**a**) which is on the GICC05 time scale^36^; background colours are with reference to Lake Suigetsu: light blue marks the first phase of the YDS, light green the transition interval and blue the second phase of the YDS.

The common timing of the bi-partition in European records, Greenland, and that of Lake Suigetsu (Fig. 3) suggests a link between the North Atlantic and Asian climatic changes. Changes in the westerlies and in North Atlantic sea surface temperatures (SST) are an important control on moisture in arid central Asia^26^ from where the dust in Greenland is derived^27^. The northward shift of the westerlies and the increased SSTs in the second phase of the YDS would have enhanced moisture transport towards central Asia. Thus, the reduced dust flux in Greenland^9^,^11^ (Fig. 2a) could be explained through an increased resistance to erosion in the source region as the result of higher moisture^28^. The increased moisture transport would likely also have increased the snow cover in Eurasia. It has been shown that changes in snow cover over Eurasia generally^29^ and over European Russia specifically^30^ can influence atmospheric circulation and thus can lead to altered dust pathways in the atmosphere and/or increased rainout of dust. An increase in snow cover can also impact the monsoonal system, specifically strengthening the EAWM^31^ and weakening the EASM^29^, in agreement with our reconstruction of the EAM. While the effect of snow cover is complex and has so far only been investigated in the present-day climate, it provides a likely explanation of how a strengthened AMOC could have reduced the dust flux to Greenland, produced a colder climate in East Asia, and thus led to the intercontinental bi-partition of the YDS and the climatic dipole between Europe and East Asia.

## Methods

### Initial Sampling and Composite Model

The cores were split in the field and core photographs were taken under natural daylight and include a depth scale and a colour chart alongside the core. Based on the core photographs the composite model was created. The uncertainty of the composite depth is the standard reading error of 1 mm (i.e. one scale unit). Afterwards, the cores were sampled using LL-channels. The LL-channels were then distributed for further analysis to the respective members of the ‘Suigetsu Varves 2006’ project. A detailed description of the coring and initial sampling can be found in ref. 17.

### Microfacies Analysis

Microfacies analysis was based on visual evaluation of thin section scans^19^ and core photos. The thin section scans were made using a slide scanner with polarising foils. The resolution used was 1,200 dpi. Additionally, seasonal layer frequency data were generated through light microscopy. These data were measured using a petrographic microscope which was equipped with a calibrated ocular micrometer, using magnifications from 25× to 400×. Layer thickness and position measurements were made mainly at 25×. Therefore, the standard reading error is 0.04 mm.

### μXRF Analysis

For μXRF analysis continuous measurements were made with an ITRAX core scanner on the sediment in LL-channels. For the Suigetsu sediment a 4 × 0.1 mm rectangular beam was used, with a step-size of 60 μm, a count time of 4 s, a voltage of 30 kV, a current of 30 mA and a Mo X-ray tube. A more detailed description of the settings used is given by^15^.

### Pollen Analysis and Modern Analogue Technique

Samples were taken contiguously with 1 cm resolution and then treated by the method of^32^. Pollen grains were identified by light microscopy and counted until the sum of the 32 arboreal pollen taxa which were identified as the most representative of the vegetation and climate^33^ exceeded 400 grains. Precision/stability of the pollen data were monitored by the standard sample method outlined by^34^.

Pollen percentage data were converted into climate indices using the modern analogue method^20^. Polygon software (http://polsystems.rits-palaeo.com) was used for calculations at the default setting of the software, i.e. number of modern analogues = 0 to 8, chord distance of threshold = 0.1; no enhancement of minor taxa. No data were rejected as outliers at this setting.

### Nitrogen Isotope Analysis on Bulk Organic Matter

Cylindrical samples with a 1 cm diameter were taken with a ≈15 cm resolution. Dried sediments were powdered and acidified within Sn cups to remove carbonate traces. ^15^N/^14^N ratios were measured using a Fisons NA 1500 CHN analyser coupled to a Finnigan-MAT Delta Plus isotope ratio mass spectrometer. Isotope ratios were calibrated against histidine, in addition to an in-house standard (marine sediment), with a reproducibility of ±0.3‰. A more detailed description of this methodology is given by^21^.

## Additional Information

**How to cite this article:** Schlolaut, G. *et al*. Evidence for a bi-partition of the Younger Dryas Stadial in East Asia associated with inversed climate characteristics compared to Europe. *Sci. Rep.* **7**, 44983; doi: 10.1038/srep44983 (2017).

**Publisher's note:** Springer Nature remains neutral with regard to jurisdictional claims in published maps and institutional affiliations.

## Supplementary Material

Supplementary Information

## Figures and Tables

**Figure 1:**
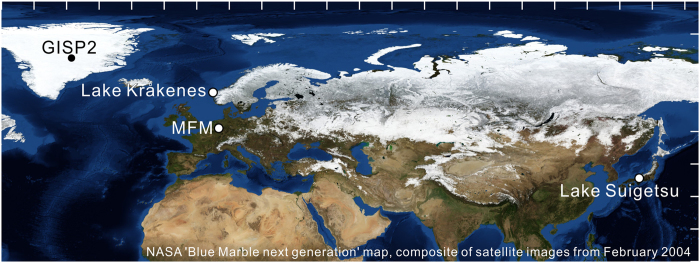
Location of Lake Suigetsu and sites referenced in the main text. Background map by NASA^35^, latitude and longitude scale resolution is 10°.

**Table 1:** Position in composite depth (cd) and age of the bi-partition in the individual proxies analysed here.

Proxy	mid YDS transition at	
	depth [cm cd]	age [SG 06_2012_ ka BP]
*Encyonema* layer frequency	1478.0 ± 0.1	12.01 ± 0.05
Mn and Mn/Fe (μXRF)	1479.4 ± 0.1	12.02 ± 0.05
pollen based temperature reconstruction	1490.9–1478.7 ± 0.6	12.14–12.02 ± 0.06
visual microfacies analysis	1498.9 ± 0.1	12.22 ± 0.04
pollen based winter precipitation reconstruction	1499.0 ± 0.6	12.22 ± 0.05

For boundary determination see Fig. 2 (more details are given in the supplement).
