# Supplementary material for: Evidence for a bi-partition of the Younger Dryas Stadial in East Asia associated with inversed climate characteristics compared to Europe
Source: Sci Rep. 2017 Mar 31;7:44983. doi: 10.1038/srep44983 (PMC5374484; doi:10.1038/srep44983)
Supplement: Supplementary Information [file srep44983-s1.pdf]

Supplement Materials for
'Evidence for a bi-partition of the Younger Dryas Stadial in East Asia associated with inversed climate characteristics compared to Europe', Nature Scientific Reports

Gordon Schlolaut*, Achim Brauer, Takeshi Nakagawa, Henry F. Lamb, Jonathan J. Tyler, Richard A. Staff, Michael H. Marshall, Christopher Bronk Ramsey, Charlotte L. Bryant, Pavel E. Tarasov

* To whom correspondence should be addressed. E-mail: gosch@jamstec.go.jp

Additional Figures:

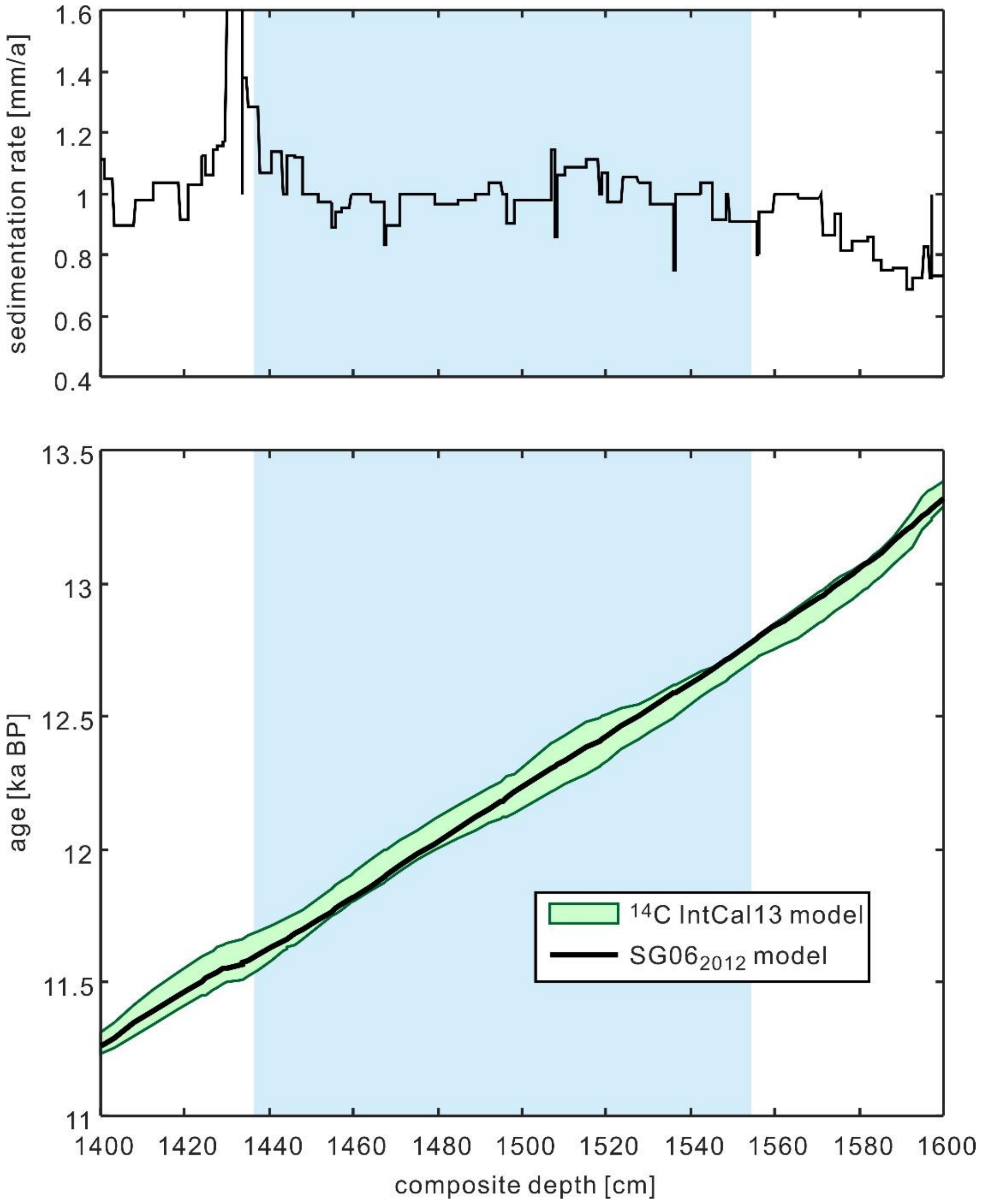

Fig. S1: top panel: sedimentation rate of the SG06$_{2012}$ age model; bottom panel: comparison between the SG06$_{2012}$ age model and the Suigetsu ^{14}C based age model calibrated using IntCal13 (Reimer et al., 2013) and modelled using OxCal ver 4.2 (https://c14.arch.ox.ac.uk/oxcal/OxCal.html). Both plots illustrate that the sedimentation rate is relatively constant during the YDS.

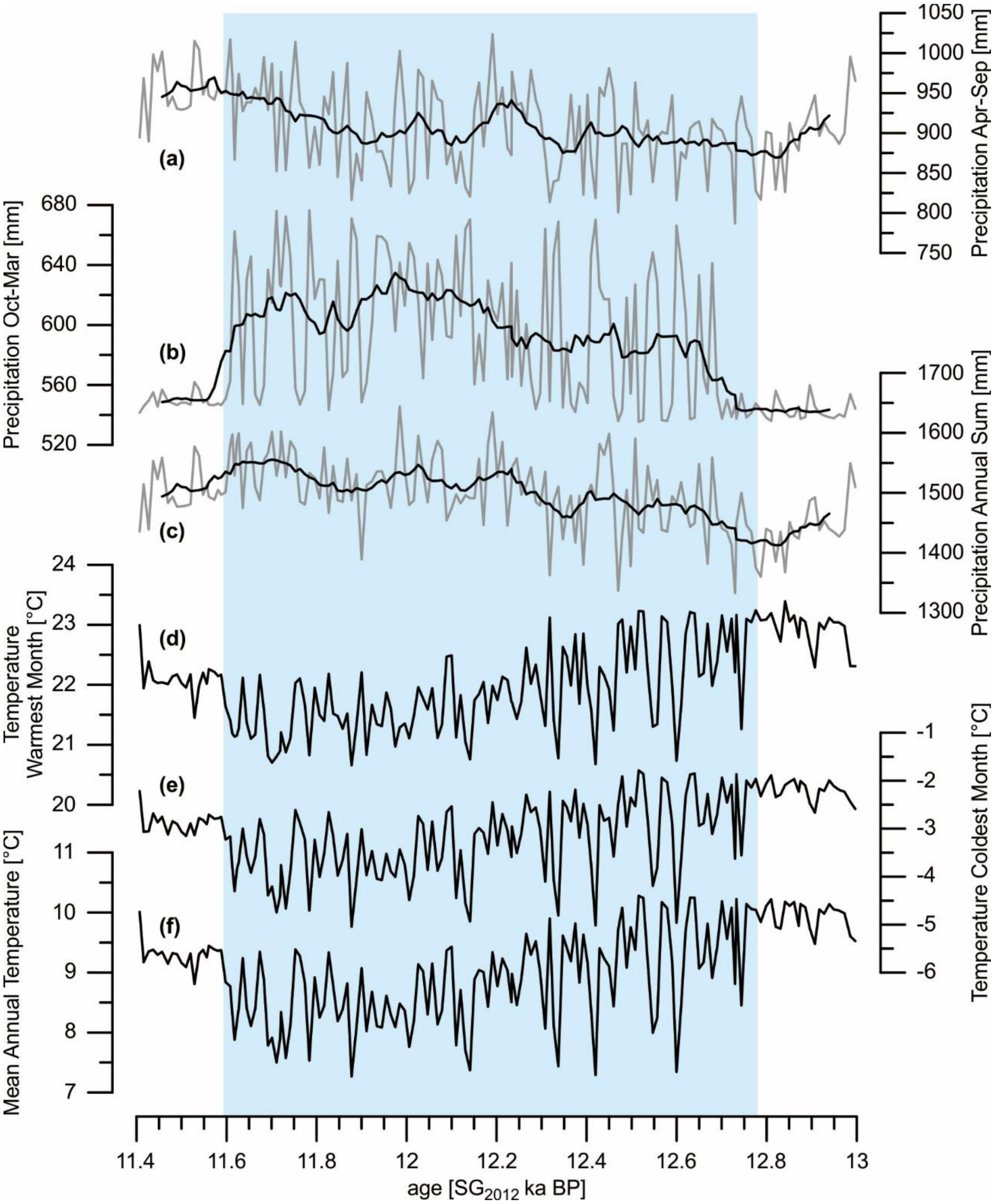

Figure S2: Pollen-based, quantitative reconstruction of the YDS climate. Top panels (a-c) show the precipitation reconstructions and that mean annual precipitation was increased (c). Seasonal precipitation reconstructions (b, c) show that this is primarily the result of an increase in winter precipitation. Bottom panels (d-f) show that there is no major difference between the seasonal and annual trend in the temperature reconstructions.

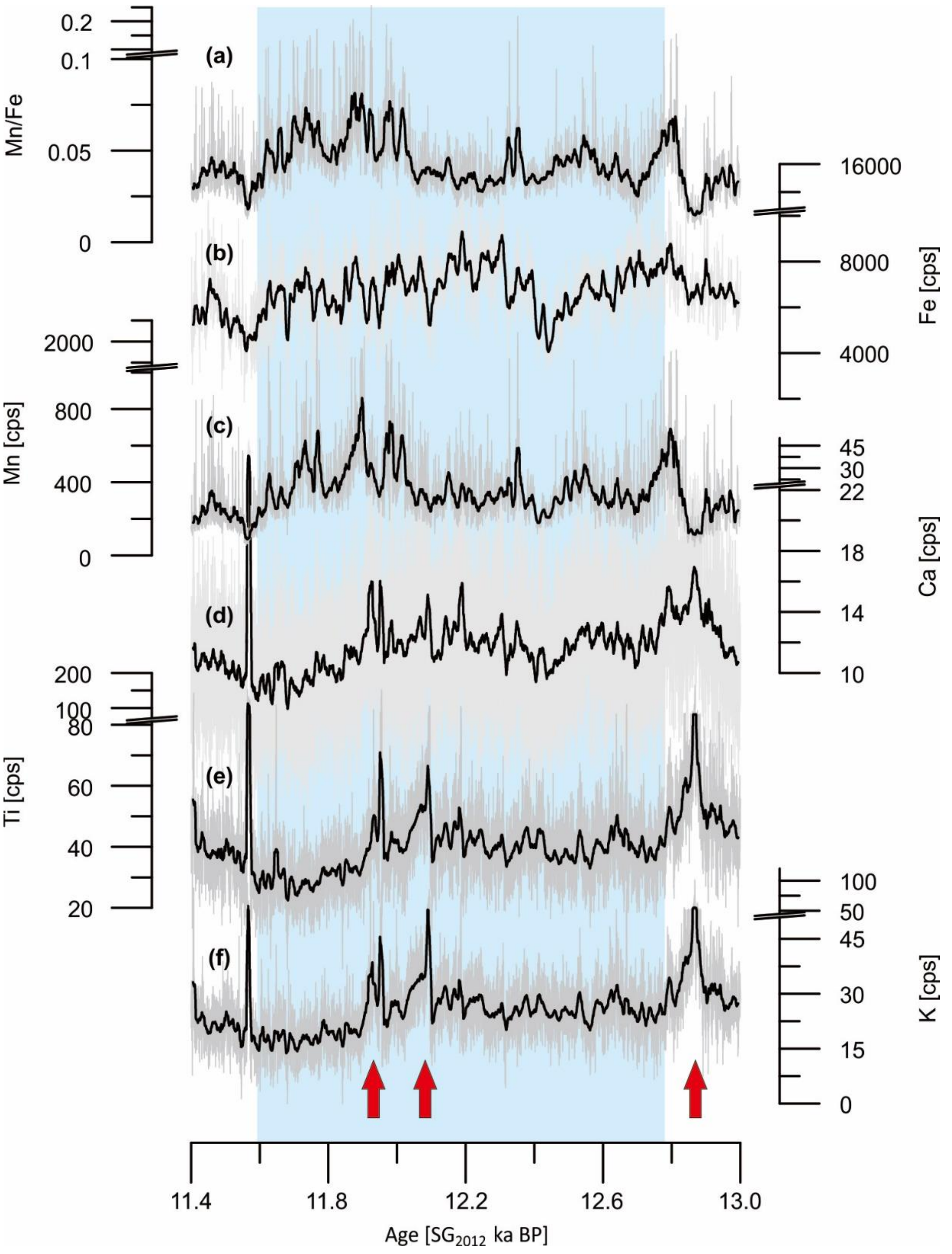

Figure S3: Graphs of all mentioned elements measured by µXRF; Mn/Fe (a), Fe (b) and Mn (c) data illustrate that Mn is the primary driver of the Mn/Fe ratio. At the bottom the K data (f) are shown together with the Ti data (e), illustrating the good correlation of the two curves. Red arrows indicate the position of the flood layers referenced in the supplementary text 'Controls on the Mn/Fe ratio at Lake Suigetsu'.

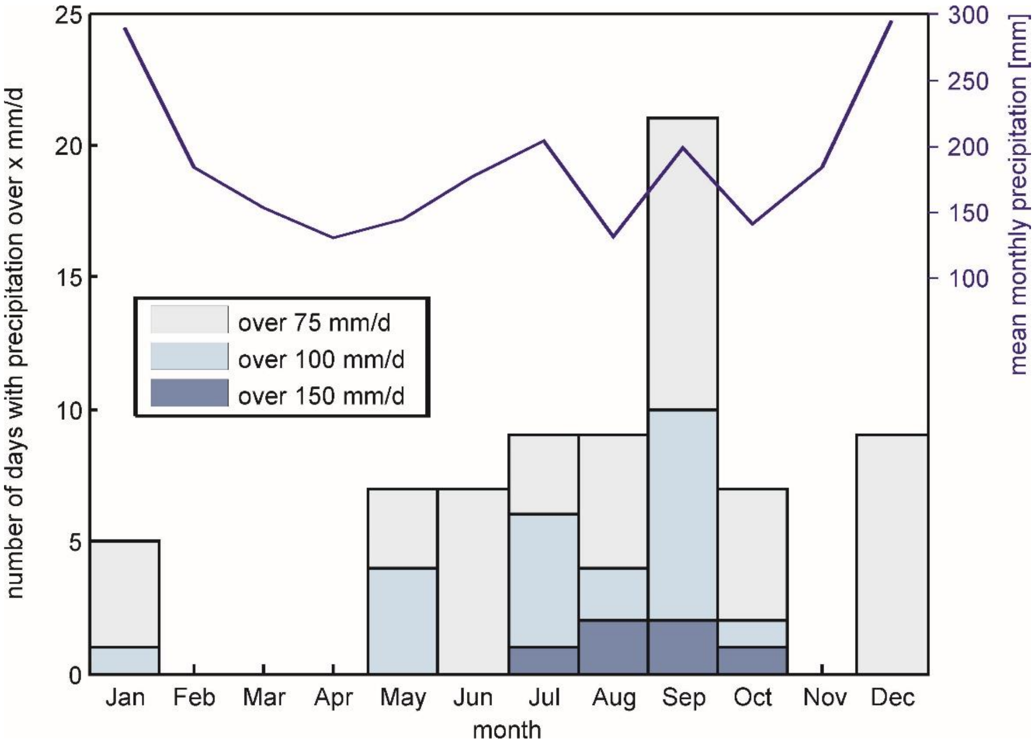

Figure S4: Precipitation values from Tsuruga station (≈20 km East of Lake Suigetsu) for the period from 1/Jan/1960 to 31/Dec/2012 (24h data integrity 82%; data source: NOAA). The data illustrate that heavy precipitation (other than snow) at Lake Suigetsu is primarily due to typhoons, for which the peak season is Aug/Sep. High mean monthly precipitation also occurs in Jun/Jul and is associated with the summer monsoon rainy season.

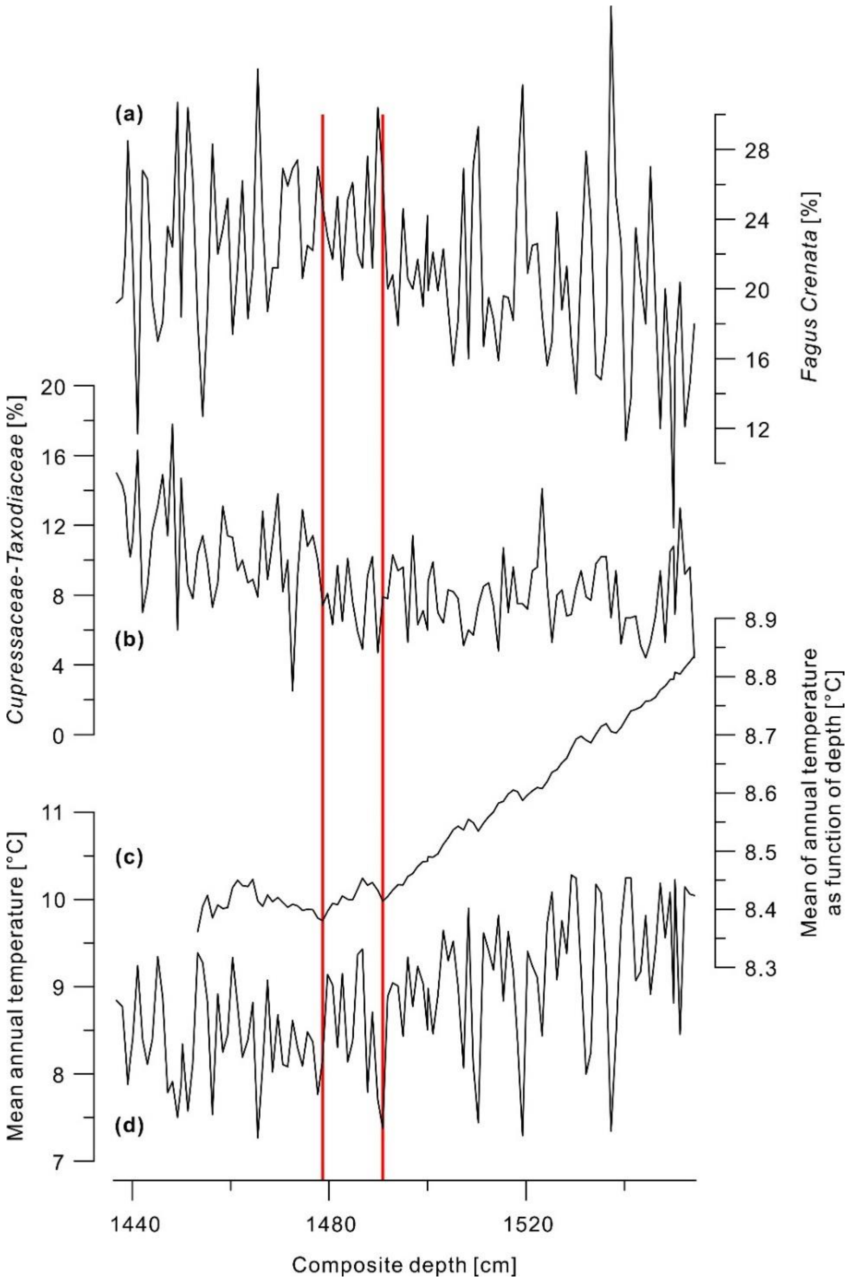

Fig. S5: Boundary determination in pollen based temperature reconstruction; (a) *Cupressaceae-Taxodiaceae* pollen concentration, (b) *Fagus crenata* pollen concentration, (c) mean of the annual temperature reconstruction for an interval beginning at the upper boundary of the YDS and ending at the respective value of the x-axis, i.e. the y-axis value at a certain depth is the mean of the data from the top of the YDS interval to this depth, (d) annual temperature reconstruction; the interval shown is from the top to the base of the YDS; (for discussion see supplement text 'Determining the boundaries between first and second phase in the individual proxies')

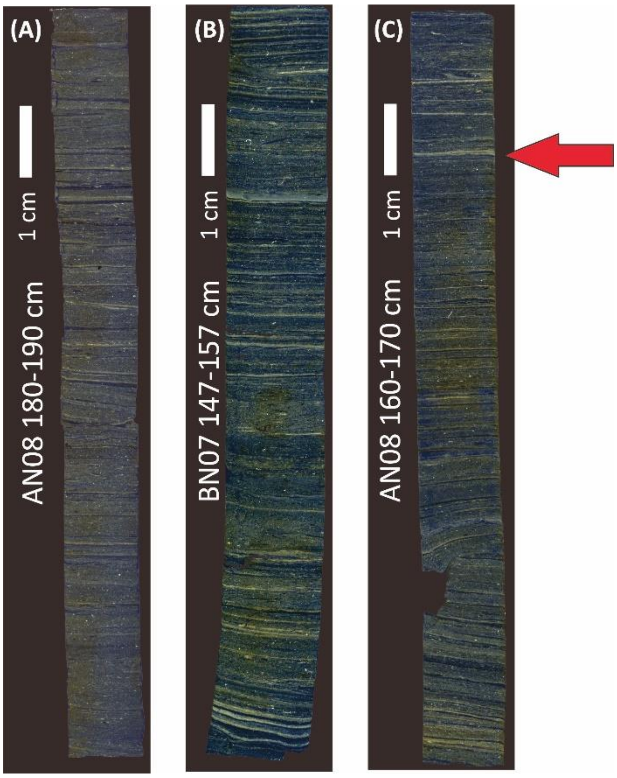

Figure S6: Thin section scans in polarised light illustrating the difference in microfacies between (A) the first phase of the YDS and (B) the second phase with its considerably more distinct siderite layers. (C) shows the point of change in microfacies (marked with a red arrow)

Additional Text:

Determining the boundaries between first and second phase in the individual proxies

Some of the proxies show relatively sudden and well defined changes, in particular the Mn/Fe ratio and the *Encyonema* layer frequency, which require no further in depth discussion.

Pollen based climate reconstruction (temperature and winter precipitation) shows a clear bi-partition, but the higher noise:signal ratio makes distinguishing boundaries less obvious. In the case of the winter precipitation reconstruction, the moving average reveals that values begin to increase from 1499 cm cd onwards, following rather constant average values in the first phase of the YDS (Fig. S2). Within the second phase of the YDS, precipitation first increases and later decreases, but remains higher than during the first phase.

The temperature reconstruction is characterised by a decreasing trend in the first phase of the YDS, followed by an approximately constant trend in the second phase. To better distinguish the change, we plotted the mean of the temperature for intervals beginning at the top of the YDS and ending at successively higher depths (Fig. S5c). The plot reveals that until 1490.9 cm cd the mean decreases with time (or increases with depth from this point downwards). However, it could also be argued that the decreasing trend in the mean temperature lasts until 1478.7 cm cd, being only briefly interrupted above 1490 cm cd. Either boundary coincides with changes in individual pollen taxa, for instance a stabilisation at high concentrations of *Fagus crenata* (1478.7 cm cd) and an increase in *Cupressaceae-Taxodiaceae* (1490 cm cd) (Fig. S5). Since there is support for either boundary, we decided to express the uncertainty as a temperature transition phase (1490 – 1478.7 cm cd).

The change in microfacies (Fig. S6) was visually determined as the point from which more pronounced siderite layers are regularly distinguishable without a return to the characteristics

of the first phase of the YDS. The frequency of more pronounced siderite layers increases with time (i.e. with decreasing depth), but it is not possible to distinguish an upper boundary for the transition visually.

The remaining two proxies are not suitable for determining the boundary between the first and second phase of the YDS since the Ti, K and Ca signals are overprinted by detrital event layers at the transition and the $\delta^{15}N$ data are too low resolution for a precise depth determination.

Controls on the Mn/Fe ratio at Lake Suigetsu

The Mn/Fe ratio of siderite in lake sediments is controlled through redox conditions and/or input of Mn and Fe into the lake (Davison, 1993; Naeher et al., 2013). In the case of Lake Suigetsu, we can observe on multiple occasions within the Last Glacial Interglacial Transition (LGIT) that enhanced siderite formation is accompanied by an increase in the Mn/Fe ratio (primarily driven by Mn – see also Figure S3) and an increase in detrital flux. Therefore, we conclude that inwash into the lake is the primary driver of the Mn/Fe ratio during the LGIT. Lake Suigetsu can be expected to be particularly susceptible to this control on the Mn/Fe ratio since the hinterland is highly enriched in Mn, as is evidenced by historic Mn mines in the vicinity of the lake.

With respect to the increased Mn/Fe ratios in the second part of the YDS it must be pointed out that two flood related event layers occur within the transition interval between the YDS phases (Fig. S3), which may have contributed to the increased Mn/Fe ratio to some degree by increasing the inwash of Mn. To illustrate the effect flood layers can have on the Mn/Fe ratio: directly before the onset of the YDS another flood layer occurs (Fig. S3) and it can be seen how the Mn/Fe ratio is increased for multiple decades after the flood. That the increase in the Mn/Fe ratio in the second phase of the YDS is not solely due to the flood layers is evidenced by the fact that the change in microfacies (Fig. S6) occurs before the first flood layer (Fig. S3, Table 1) and by the

fact that the changes last too long (i.e. several centuries) to be solely the result of Mn brought into the lake during these flood events.

However, the redox conditions are also an important driver for siderite formation at Lake Suigetsu, especially for the high frequency changes in the Mn/Fe ratio. During the second part of the YDS (where Mn and Mn/Fe values are high and variations clearly distinguishable) the high frequency changes of the Mn/Fe ratio are correlated with the frequency of seasonal siderite layers, as well as with the frequency of seasonal layers of *Aulacoseira* spp. diatoms and layers of light amorphous organic material. All of these layers have been related to seasonal stratification and lake overturn, respectively (Schlolaut et al., 2012), which in turn exert a strong control on the redox conditions in the lake. Therefore, we conclude that the high frequency changes in the Mn/Fe ratio reflect changes in redox conditions. However, while the high frequency changes do correlate between the Mn/Fe ratio and the seasonal layer frequencies, there is no strong indication of the bi-partition in the seasonal layer frequencies. Therefore, we exclude a strong influence of the redox conditions on the observed bi-partition of the YDS in the Mn/Fe ratio. Explicitly we also exclude that the enhanced siderite formation is the result of a stronger stratification as the result of an increase in lake level, considering that the lake has an outflow, that would need to be blocked to facilitate a rise in lake level. While blocking as the result of tectonic processes is possible and has occurred in the past (see Schlolaut et al., 2014 and references therein), it is unlikely that tectonically controlled lake level changes coincide exactly with the second phase of the YDS and the hypothesis conflicts with the $\delta^{15}N$ and *Encyonema* data. Furthermore, there are no deposits or sediment structures suggesting any major fault movement during the YDS.

µXRF measurements of Ti, K and Ca as tracers for the detrital fraction

Major episodes of increased detrital flux in the Lake Suigetsu sediment (e.g. event layers, but also core intervals rich in diffuse detrital material) are easily identifiable in core photos and thin sections and can be correlated to high values in Ti, K and Ca values, thus showing that these µXRF data can be used as proxies for the detrital fraction in the sediment. However, changes in the µXRF data which coincide with facies changes or which are not very pronounced (as with the decrease in Ti, K and Ca in the second phase of the YDS) must be viewed with some caution since there are other influences possible. A common issue with µXRF data from sediment cores is variable water content. But in the case of the drop in Ti, K and Ca in the second phase of the YDS the signal is reproduced on resin impregnated (i.e. dry) samples, thus eliminating the possibility of an artefact by variable water content. The near constant sedimentation rates during the YDS (Fig. S1) and the lack of any correlation between the element curves and the sedimentation rate mean that dilution of detrital material is not occurring. We also rule out overprinting of the detrital signal by the enhanced siderite formation by coating of detrital grains since the high frequency changes in the Mn/Fe ratio are not mirrored in the Ti, K and Ca curves. Thus we conclude that the changes observed in the Ti, K and Ca curves are indeed reflecting a decrease in detrital material in the sediment. With respect to Ca it is important to note that calcium carbonate formation was not observed in the sediment and the data thus not biased in this regard.

References:

Davidson, W., 1993. Iron and manganese in lakes. Earth Science Reviews 34, 119 – 163.

Naeher, S., Gilli, A., North, R.P., Hamann, Y., Schubert, C.J., 2013, Tracing bottom water oxygenation with sedimentary Mn/Fe ratios in Lake Zurich, Switzerland. Chemical Geology 352, 125 – 133.

Reimer, P.J., Baillie, M.G.L., Bard, E., Bayliss, A., Beck, J.W., Blackwell, P.G., Bronk Ramsey, C., Buck, C.E., Burr, G.S., Edwards, R.L., Friedrich, M., Grootes, P.M., Guilderson, T.P., Hajdas, I., Heaton, T.J., Hogg, A.J., Hughen, K.A., Kaiser, K.F., Kromer, B., McCormac, F.G., Manning, S.W., Reimer, R.W., Richards, D.A., Southon, J.R., Talamo, S., Turney, C.S.M., van der Plicht, J., Weyhenmeyer, C.E., 2009. IntCal09 and Marine09 radiocarbon age calibration curves, 0-50,000 years cal BP. Radiocarbon 51, 1111 – 1150.

Schlolaut, G., Brauer, A., Marshall, M.H., Nakagawa, T., Staff, R.A., Bronk Ramsey, C., Lamb, H.F., Bryant, C.L., Naumann, R., Dulski, P., Brock, F., Yokoyama, Y., Tada, R., Haraguchi, T., Suigetsu 2006 project members, 2014. Event layers in the Japanese Lake Suigetsu `SG06' sediment core: description, interpretation and climatic implications. Quaternary Science Reviews 83, 157 – 170.

Schlolaut, G., Marshall, M.H., Brauer, A., Nakagawa, T., Lamb, H.F., Staff, R.A., Bronk Ramsey, C., Bryant, C.L., Brock, F., Kossler, A., Tarasov, P.E., Yokoyama, Y., Tada, R., Haraguchi, T., Suigetsu 2006 project members, 2012. An automated method for varve interpolation and its application to the Late Glacial chronology from Lake Suigetsu, Japan. Quaternary Geochronology 13, 52 – 69.
